# Inhibition of Nrf2/HO-1 signaling leads to increased activation of the NLRP3 inflammasome in osteoarthritis

**DOI:** 10.1186/s13075-019-2085-6

**Published:** 2019-12-23

**Authors:** Zhuming Chen, Huan Zhong, Jinsong Wei, Sien Lin, Zhixian Zong, Fan Gong, Xinqia Huang, Jinhui Sun, Peng Li, Hao Lin, Bo Wei, Jiaqi Chu

**Affiliations:** 1 Orthopedic Center, Affiliated Hospital of Guangdong Medical University, Zhanjiang, 524001 China; 2 Department of Gastroenterology, Affiliated Hospital of Guangdong Medical University, Zhanjiang, 524001 China; 3 Stem Cell Research and Cellular Therapy Center, Affiliated Hospital of Guangdong Medical University, Zhanjiang, 524001 China

**Keywords:** Osteoarthritis, Synovitis, NLRP3, ROS, Nrf2/HO-1 signaling

## Abstract

**Introduction:**

Osteoarthritis (OA) is an inflammatory disease of the joints that causes progressive disability in the elderly. Reactive oxygen species (ROS) play an important role in OA development; they may activate the NLRP3 inflammasome, thereby inducing the secretion of proinflammatory IL-1β and IL-18, leading to the aggravation of the downstream inflammatory response. Nrf2 is a key transcription factor that regulates the expression of antioxidant enzymes that protect against oxidative stress and tissue damage. We aimed to explore the underlying mechanism of OA development by investigating NLRP3, ASC, Nrf2, and HO-1 expression in synovia and their regulatory networks in OA.

**Methods:**

Human total knee replacement samples were subjected to histology and micro-CT analysis to determine the pathological changes in the cartilage and subchondral bone and to assess the expression of inflammation-related markers in the synovial tissue by immunohistochemistry (IHC), qRT-PCR, and Western blot. To investigate these pathological changes in an OA animal model, adult Sprague-Dawley rats were subjected to anterior cruciate ligament transection and medial meniscectomy. Articular cartilage and subchondral bone changes and synovial tissue were also determined by the same methods used for the human samples. Finally, SW982 cells were stimulated with lipopolysaccharide (LPS) as an in vitro inflammatory cell model. The correlation between NLRP3 and Nrf2 expression was confirmed by knocking down NLRP3 or Nrf2.

**Results:**

Cartilage destruction and subchondral bone sclerosis were found in the OA patients and OA model rats. Significantly increased expression levels of NLRP3, ASC, Nrf2, and HO-1 were found in the synovial tissue from OA patients. NLRP3, ASC, Nrf2, and HO-1 expression in the synovium was also upregulated in the OA group compared with the sham group. Furthermore, the NLRP3, Nrf2, HO-1, IL-1β, and IL-18 expression in LPS-treated SW982 cells was increased in a dose-dependent manner. As expected, the expression of NLRP3 was upregulated, and the expression of IL-1β and IL-18 was downregulated after Nrf2 silencing. However, knocking down NLRP3 did not affect the expression of Nrf2.

**Conclusions:**

ROS-induced oxidative stress may be the main cause of NLRP3 inflammasome activation and subsequent release of downstream factors during OA development. Nrf2/HO-1 signaling could be a key pathway for the activation of the NLRP3 inflammasome, which may contribute to the progression of OA. Herein, we discovered a novel role of Nrf2/HO-1 signaling in the production of NLRP3, which may facilitate the prevention and treatment of OA.

## Background

Osteoarthritis (OA), characterized by progressive cartilage degeneration and secondary synovial inflammation, is one of the most common chronic joint diseases affecting people of all ages, especially the elderly, and causes severe pain and physical disability [1]. Conservative non-surgical management of OA involves physical interventions (e.g., weight reduction, moderate exercises, and stretching) and pharmacological therapies including nonsteroidal anti-inflammatory drugs, opioid analgesics, and intra-articular administration of steroids and hyaluronic acid, which is effective in alleviating pain but insufficient to reverse cartilage damage [2]. If patients suffer from severe joint damage or OA fails to respond to the conservative management, surgical treatments such as arthroplasty and osteotomy are generally recommended. However, the long-term outcome in surgery patients differs significantly [3]. Previously OA was considered a noninflammatory arthropathy, but today, it is generally accepted that it is an inflammatory disease, with synovitis present in most patients. Proinflammatory cytokines (e.g., interleukin-1 beta (IL-1β), and IL-18), reactive oxygen species (ROS), inflammatory mediators, and biomechanical stress are regarded to be the major factors contributing to this scenario [4]. Furthermore, recent studies have demonstrated that dysregulated microRNAs, obesity-related metabolic factors, and inflammasome signaling molecules including Nod-like receptor protein 3 (NLRP3) are also involved in the progression of OA [5–8].

NLRP3, along with the adaptor protein apoptosis-associated speck-like protein containing a caspase recruitment domain (ASC), is one of the most studied inflammasome sensors, which activates caspase-1 via the assembly of a complete inflammasome complex, subsequently leading to the secretion of pro-inflammatory cytokines IL-1β, IL-18, and tumor necrosis factor alpha [9, 10]. These play crucial roles in OA pathogenesis resulting in the release of cartilage-degrading enzymes, such as aggrecanases and metalloproteinases, from chondrocytes [11, 12]. Current ligands (known as NLRP3 agonists) that induce NLRP3 inflammasome formation include ATP, pore-forming toxins, crystalline substances, nucleic acids, hyaluronan, and fungal, bacterial, and viral pathogens [13]. Likewise, recent studies suggest that intracellular ROS is elevated by various NLRP3 stimulators and enhanced ROS is essential for NLRP3 inflammasome activation [14]. The nuclear factor E2-related factor 2 (Nrf2) plays an important role in regulating the expression of antioxidant proteins, such as heme oxygenase 1 (HO-1), and is activated in response to ROS to protect cells against oxidative stress triggered by inflammation [15]. However, the relationship between NLRP3, ROS, and Nrf2 has not been determined.

In the present study, we addressed the question of whether the Nrf2 antioxidative pathway suppressed NLRP3-mediated proinflammatory responses in OA. To answer this question, we compared the expression changes of key molecules belonging to Nrf2 signaling and NLRP3 inflammasome pathway, using articular cartilage and synovial tissues obtained from both OA patients and a surgically induced rat model of OA. In addition, the relationship between NLRP3, ROS, and Nrf2 was explored in vitro through gene-specific inhibition of NLRP3 and Nrf2, respectively.

## Methods

### Clinical specimen harvest

We collected cartilage and synovium samples from OA patients during total knee arthroplasty surgery (*n* = 12; 4 men and 8 women; age 66.4 ± 7.2 years, ranging from 56 to 78 years). The clinical samples for the control (CTL) group were collected from patients with no previous history of OA whose knees were amputated due to trauma (*n* = 5; 1 man and 4 women, aged 50.0 ± 23.4 years, ranging from 15 to 79 years). All patients satisfied the American College of Rheumatology clinical criteria for primary knee OA [16]. Samples of synovium and cartilage were collected within half an hour of the surgery, stored into phosphate-buffered saline, and transported to the laboratory within 15 min. Half of each specimen was fixed in formalin for later paraffin embedding, and the other half of each sample was frozen immediately in liquid nitrogen for use in IHC and Western blot analyses and RNA extraction. All clinical procedures were approved by the Committee of Medical Ethics at the Affiliated Hospital of Guangdong Medical University (PJ2017072; Sept 7, 2017). The clinical specimens (cartilage and synovium) were obtained from donors after they signed an informed consent form.

### Rat models of osteoarthritis and tissue harvest

Four-month-old Sprague-Dawley (SD) rats were purchased from the Guangdong Medical Laboratory Animal Center of China. Rats were housed with free access to pelleted commercial food and water and were individually housed with a 12-h dark/light cycle under controlled conditions (25 °C, 70% humidity). Animals were randomly allocated to the sham group (*n* = 8) or OA group (*n* = 16). For the OA model, the right knee joints of the rats were subjected to both anterior cruciate ligament transection and medial meniscus destabilization (ACLT + MMD). The sham group received only a skin and capsule incision, and samples were collected at 4 and 8 weeks after the sham operation. OA group samples were collected at 4 (*n* = 8) or 8 (*n* = 8) weeks after operations. All protocols involving animals were approved by the Animal Care and Experiment Committee of Guangdong Medical University and followed the National Institutes of Health guidelines for the Care and Use of Laboratory Animals.

### Cell culture

The SW982 cell line was purchased from the American Type Culture Collection (ATCC, Manassas, VA, USA), and the cells were cultured in Dulbecco’s modified Eagle’s medium (DMEM) containing 10% fetal bovine serum (FBS) and an antibiotic-antimycotic solution (all from Gibco, Grand Island, NY, USA), at 37 °C in humidified conditions with 5% CO_2_. The medium was changed every 3 days. Cells were treated with 0, 50, 100, and 200 ng/ml LPS (Sigma, St. Louis, MO, USA) in a 10-cm Petri dish for 24 h.

### siRNA transfection

SW982 cells were transfected in 6-well plates with Nrf2-specific siRNA using Lipofectamine® LTX & PLUS™ Reagent (Life Technologies, Carlsbad, CA, USA) in accordance with the manufacturer’s instructions. The expression of Nrf2 and downstream proteins was examined 48 h after transfection by using qRT-PCR and Western blotting.

### shRNA lentiviral particle transduction

The control and NLRP3 shRNA lentiviral particles were purchased from Santa Cruz Biotechnology (CA, USA). SW982 cells were seeded in a 24-well plate, and cells were infected with lentiviral particles for 72 h. Then, 4 μg/ml puromycin (Santa Cruz Biotechnology) was added to the medium to select cells successfully infected with the virus.

### Intracellular ROS detection by DHE

ROS production in SW982 cells was measured by using dihydroethidium (DHE, Santa Cruz) fluorescent dye. Cells on glass coverslips were incubated in Hank’s balanced salt solution (HBSS, Gibco) containing CaCl_2_ and MgCl_2_ with 10 μmol/L DHE in a light-protected chamber at 37 °C for 30 min. Then, the cells were washed with HBSS and loaded onto microscope slides using a mounting medium containing DAPI (Vector Laboratories, CA, USA), and fluorescence microscopy was used to capture images of the cells. In addition, ROS levels were determined by flow cytometry at an excitation wavelength of 535 nm and an emission wavelength of 610 nm. All experiments were performed in triplicate.

### Micro-computed tomography (CT) analysis

Articular cartilage tissues were fixed overnight in 10% formalin. The microstructure of the samples was analyzed using micro-CT (SkyScan1172; Bruker, Kontich, Belgium). For the clinical samples, the three-dimensional (3D) images showing the whole subchondral bone were used for analysis; for the animal study, 50 sagittal images of the tibia subchondral bone were evaluated at a global threshold (158 mg hydroxyapatite/cm^3^) to reconstruct a 3D model, and a Gaussian filter (sigma = 0.8, support = 2) was used to suppress noise. The bone mineral density (BMD) and bone volume/total tissue volume (BV/TV) were analyzed as 3D structural parameters with a built-in program. All of the micro-CT analyses were performed using the Scanco micro-CT software analysis system (Scanco Medical, v. 2013).

### Histological and immunochemical analysis

Articular cartilage was decalcified using 10% ethylenediaminetetraacetic acid (EDTA, Solarbio, Beijing, China) for 2 weeks and embedded in paraffin blocks. The blocks were cut to a thickness of 5 μm at distinct planes using a microtome (Leica Microsystems, Germany). Then, the sections were subjected to safranin O/fast green, Masson, hematoxylin and eosin (H&E), and immunohistochemical staining (all from Solarbio).

For immunohistochemical staining, horseradish peroxidase-streptavidin in the detection system (#CW0128, CWBIO, China) was utilized and diluted according to the appropriate ratio with the following antibodies: NLRP3 (1:100, #13158, Cell Signaling Technology, Danvers, MA, USA), ASC (1:100, #78977, Cell Signaling Technology, Danvers, MA, USA), Nrf2 (1:100, #ab62352, Abcam, USA), and HO-1 (1:100, #ADI-SPA-895, ENZO, USA). Subsequently, counterstaining was performed with hematoxylin. Photographs of the selected areas were taken using an optical microscope and quantified with Image-Pro Plus 6.0 software (Media Cybernetics, Silver Spring, MD, USA) based on the histological staining. The optical density of the positively stained cells was measured; the measurement was repeated three times in randomly selected sections of the region of interest for each sample.

For cartilage analysis and scoring, proteoglycan in the articular cartilage was measured by safranin O/green staining and blindly scored by three independent researchers based on the OARSI histologic scoring system [17]. Briefly, the depth of the cartilage damage and the extent of the surface damage was scored in a blinded manner at four different locations in the rat knee joint, i.e., the lateral and medial tibia and femur. The OA score was defined as the product of the multiplication of these two scores. The mean OA score was calculated using the scores for the four individual locations examined by three researchers.

### Gene expression analysis

For the gene expression analysis, the samples were collected, and total RNA was extracted using TRIzol reagent (Invitrogen Life Technologies, Carlsbad, CA, USA) according to the manufacturer’s instructions. Total RNA (1 μg) was reverse transcribed to cDNA using Prime Script RT Enzyme mix at 37 °C for 15 min, followed by incubation at 85 °C for 5 s. The primer sequences are shown in Table 1. GAPDH was used as an internal control. All procedures were performed with an ABI 7500 Fast Real-Time PCR system (Applied Biosystems, Carlsbad, CA, USA) under the following cycling conditions: 95 °C for 30 s, followed by 40 cycles at 95 °C for 5 s and 60 °C for 34 s. The relative quantification of the gene expression was calculated with the 2^−ΔΔCT^ values normalized to the GAPDH values. Primer sequences were determined through GenBank, as shown in Table 1, and were synthesized by Biotechnology Co. Ltd. (Shanghai).

**Table 1:** Primer sequences for real-time PCR

Gene name	Primer sequence (5′ to 3′)		Product (bp)	GenBank serial number
Nrf2	Forward	GGTTGCCCACATTCCCAAATC	119	NM_001313901.1
	Reverse	CAAGTGACTGAAACGTAGCCG		
NLRP3	Forward	AAAGGAAGTGGACTGCGAGA	129	XM_011544055.2
	Reverse	TTCAAACGACTCCCTGGAAC		
HO-1	Forward	TTCAAGCAGCTCTACCGCTC	90	NM_002133.2
	Reverse	GAACGCAGTCTTGGCCTCTT		
IL-1β	Forward	CCACAGACCTTCCAGGAGAA	121	XM_017003988.1
	Reverse	GTGATCGTACAGGTGCATCG		
IL-18	Forward	TGCATCAACTTTGTGGCAAT	169	XM_011542806.2
	Reverse	ATAGAGGCCGATTTCCTTGG		
GAPDH	Forward	GGACTCATGACCACAGTCCAT	109	NM_000194.2
	Reverse	CAGGGATGATGTTCTGGAGAG		

### Protein analysis

The protein used for Western blotting was extracted using RIPA buffer (#89900, Thermo Fisher Scientific, Grand Island, NY, USA). Then, the lysates were centrifuged at 1000×*g* for 5 min to remove tissue. Protein concentrations were determined using a BCA protein assay kit (#P0010, Beyotime Biotechnology, China). Total protein (20 μg) was separated by 10% or 12% SDS-polyacrylamide gel electrophoresis (SDS-PAGE) and transferred to polyvinylidene fluoride (PVDF) membranes (#R7EA2546B, Millipore Corp, GER). The membranes were then blocked in TBS-T containing 5% skim milk for 1 h and incubated with primary antibodies against NLRP3 (1:1000, #13158, Cell Signaling Technology), Nrf2 (1:1000, #ab62352, Abcam), P-Nrf2(1:1000, #ab76026, Abcam), HO-1 (1:500, #ADI-SPA-895, ENZO), IL-1β (1:1000, #12703S, Sigma), IL-18 (1:1000, #sab2701968, Sigma), α-tubulin (1:1000, #sc-32293, Santa Cruz), and GAPDH (1:1000, #sc-25778, Santa Cruz) overnight at 4 °C. The membranes were then washed with TBS-T and incubated with the secondary antibody at the recommended dilution in blocking buffer at room temperature for 2 h. The blots were then rinsed 3 times for 5 min with TBS-T, and images were captured using a CCD camera (Azure Biosystems C500, USA).

### Enzyme-linked immunosorbent assay (ELISA)

For clinical samples, IL-1β levels in the serum were detected using an IL-1β ELISA kit according to the protocol provided by the manufacturer (#MM-0181H1, MEIMIAN, China). The results were obtained based on the linear range of the standard curve. All of the samples were assessed in triplicate.

### Statistical analysis

All data are presented as mean ± standard deviation (SD). Statistical analysis was performed with the paired Student *t* test to compare mean values between 2 groups. One-way analysis of variance (ANOVA) with Turkey Kramer multiple comparisons test was used to compare mean values using the SPSS software (version 19.0, SPSS Inc., USA). *P* < 0.05 was considered statistically significant.

## Results

### Cartilage destruction and subchondral bone sclerosis in OA patients

**Fig. 1:**
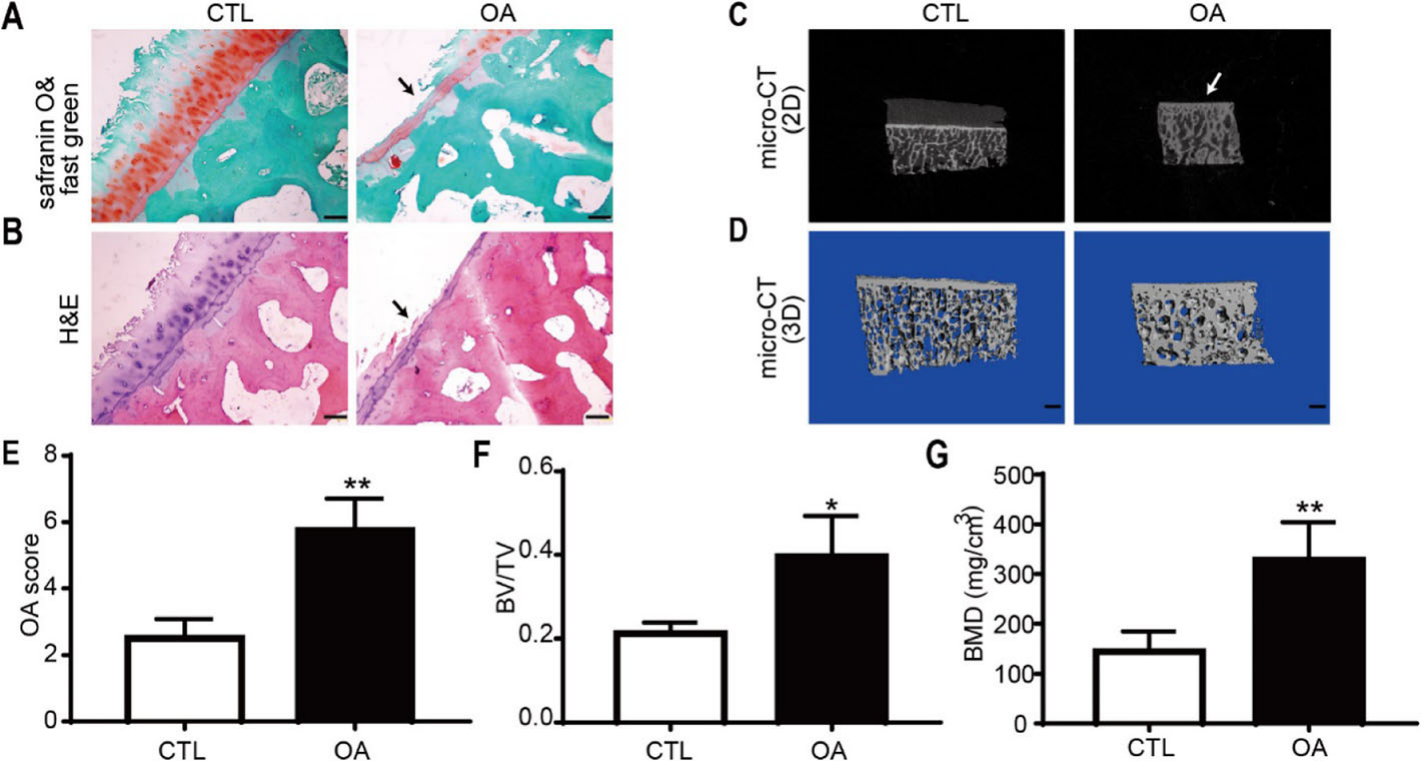
Histological and microstructural changes in the subchondral bone of the superior articular surface on the tibia. Representative images are shown from repeated experiments. **a**, **b** Safranin O/fast green- and H&E-stained sections showing the damaged region of the articular surface (indicated by black arrows; scale bar = 100 μm). **c**, **d** 2D and 3D images of the subchondral bone of the tibia showing the damaged region of the articular surface (indicated by white arrows; scale bar = 1 mm). **e** Semiquantitative results of the OARSI scoring system. **f**, **g** Bone quantification results of the subchondral bone of the tibia from healthy controls and OA patients, including the BV/TV (trabecular BV per TV) and BMD. (*n* = 4, *P < 0.05, **P < 0.01 vs CTL)

For the clinical samples, safranin O/fast green and H&E staining were used. The results showed that the surface of the cartilage was relatively intact with the appropriate amount of chondrocytes in the control group. However, there was a significant reduction in cartilage thickness and chondrocyte numbers in the OA group cartilage (Fig. 1a, b). In addition, based on the OARSI histologic grading system, compared with that in the control group (2.50 ± 0.28, *n* = 4), the cartilage damage in the OA group (5.75 ± 0.47, *n* = 4) was more severe (Fig. 1e). Furthermore, 2D images and 3D reconstructed images captured from the micro-CT analysis showed that the BMD of OA patients was significantly higher than that of the healthy controls (0.21 ± 0.01 vs 0.39 ± 0.05 BV/TV and 144.70 ± 19.81 mg/cm^3^ vs 327.50 ± 38.33 mg/cm^3^ BMD, *P* < 0.05, *n* = 4; Fig. 1c, d, f, g).

### Expression of inflammasome proteins and Nrf2/HO-1 in the OA cartilage synovium

**Fig. 2:**
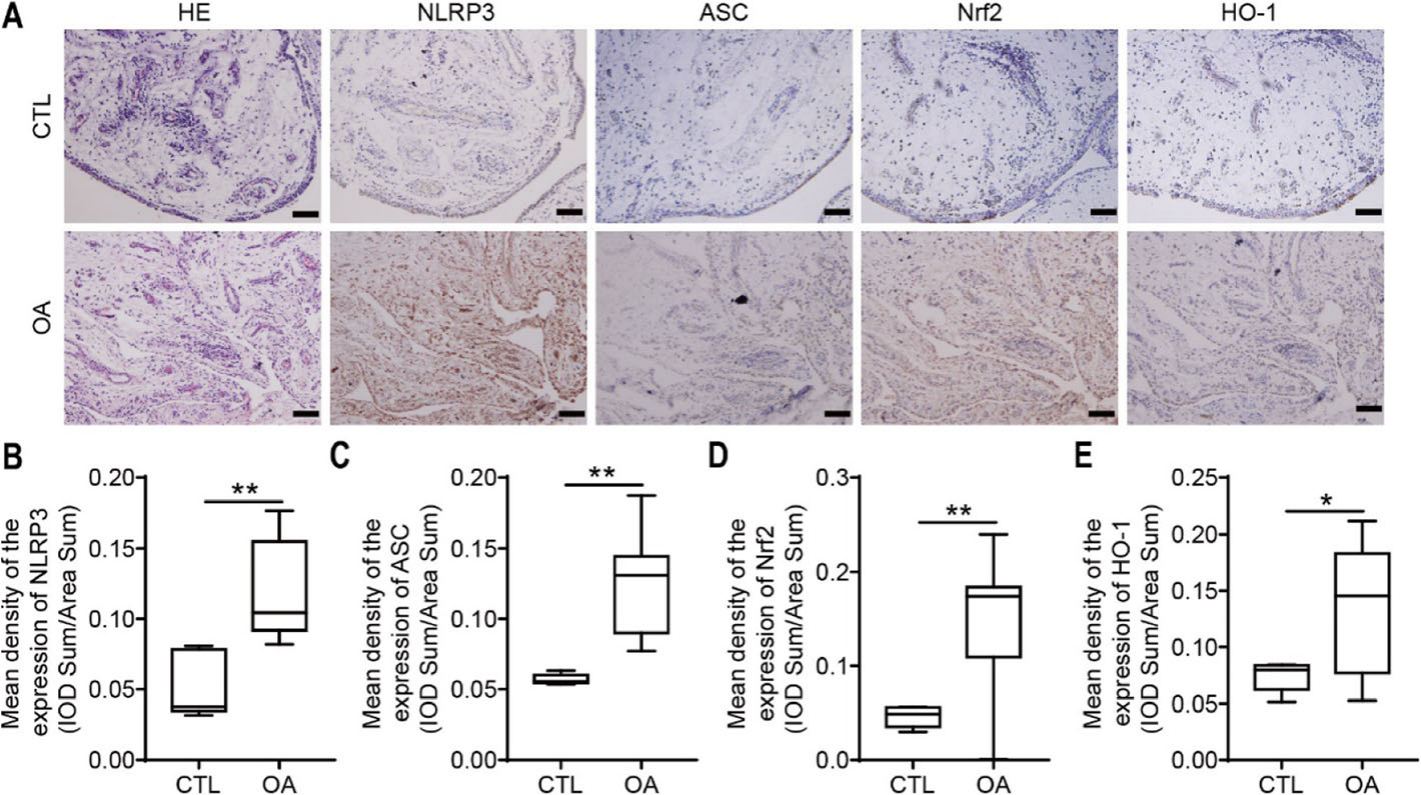
Immunohistochemistry analysis of the synovium of OA patients. Representative images are shown. **a** H&E and IHC staining of the synovium in the knee joints. Scale bar = 100 μm. **b** Relative expression level of NLRP3. **c** Relative expression level of ASC. **d** Relative expression level of Nrf2. **e** Relative expression level of HO-1 (*P < 0.05, **P < 0.01 vs CTL)

Nrf2, HO-1, NLRP3, and ASC expression levels in the synovium and the phenotype of synovium were investigated in OA patients by immunohistochemistry. Colored staining, representing the expression of Nrf2, HO-1, NLRP3, or ASC, was observed in the synovial membranes of the OA patients (Fig. 2a). Compared with that in the control group, the expression of NLRP3 and ASC in the OA group was significantly increased (0.12 ± 0.01, *n* = 12 vs 0.05 ± 0.01, *n* = 5, *P* < 0.01; 0.13 ± 0.01, *n* = 12 vs 0.06 ± 0.01, *n* = 5, *P* < 0.01; Fig. 2b, c). Moreover, the results also indicated that the expression of Nrf2 and HO-1 was increased in the OA group compared with the control group (0.15 ± 0.02, *n* = 12 vs 0.05 ± 0.01, *n* = 5, *P* < 0.01, and 0.14 ± 0.02, *n* = 12 vs 0.07 ± 0.01, *n* = 5, *P* < 0.05; Fig. 2d, e).

### Synovial NLRP3, Nrf2, and HO-1 expression, and serum inflammatory factor IL-1β levels in OA patients

**Fig. 3:**
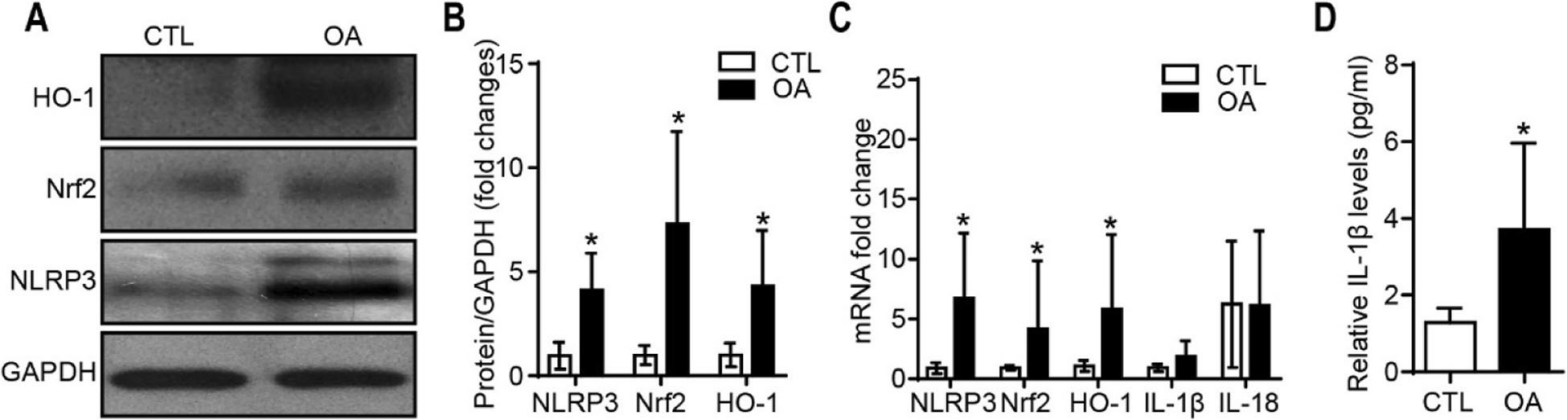
Relative protein and mRNA expression levels in the synovium and relative IL-1β level in the serum of OA patients. Representative images are shown from experiments. **a**, **b** Nrf2, NLRP3, and HO-1 expression levels were determined using Western blot analysis. **c** Nrf2, NLRP3, and HO-1 mRNA expression levels were examined using qRT-PCR. **d** Expression level of IL-1β in the serum, assessed by ELISA. (^n.s.^*P* > 0.05, *P < 0.05, **P < 0.01 vs CTL)

For clinical samples, Western blot analyses were employed to compare the expression levels of Nrf2, NLRP3, and HO-1 in OA patients with those in the healthy controls. Nrf2, NLRP3, and HO-1 expression was upregulated dramatically in OA patients (Fig. 3a, b). In addition, as shown in Fig. 3c, the mRNA fold changes of Nrf2, NLRP3, and HO-1 were also increased in the synovium as assessed by qRT-PCR, but there were no significant differences in IL-1β or IL-18 expression. However, the ELISA results showed that the level of IL-1β was significantly elevated in the serum of OA patients compared with the healthy controls (3.71 ± 0.92 pg/ml vs 1.28 ± 0.15 pg/ml, *n* = 6, *P* < 0.05; Fig. 3d).

### Cartilage and subchondral bone structure of OA animal models

**Fig. 4:**
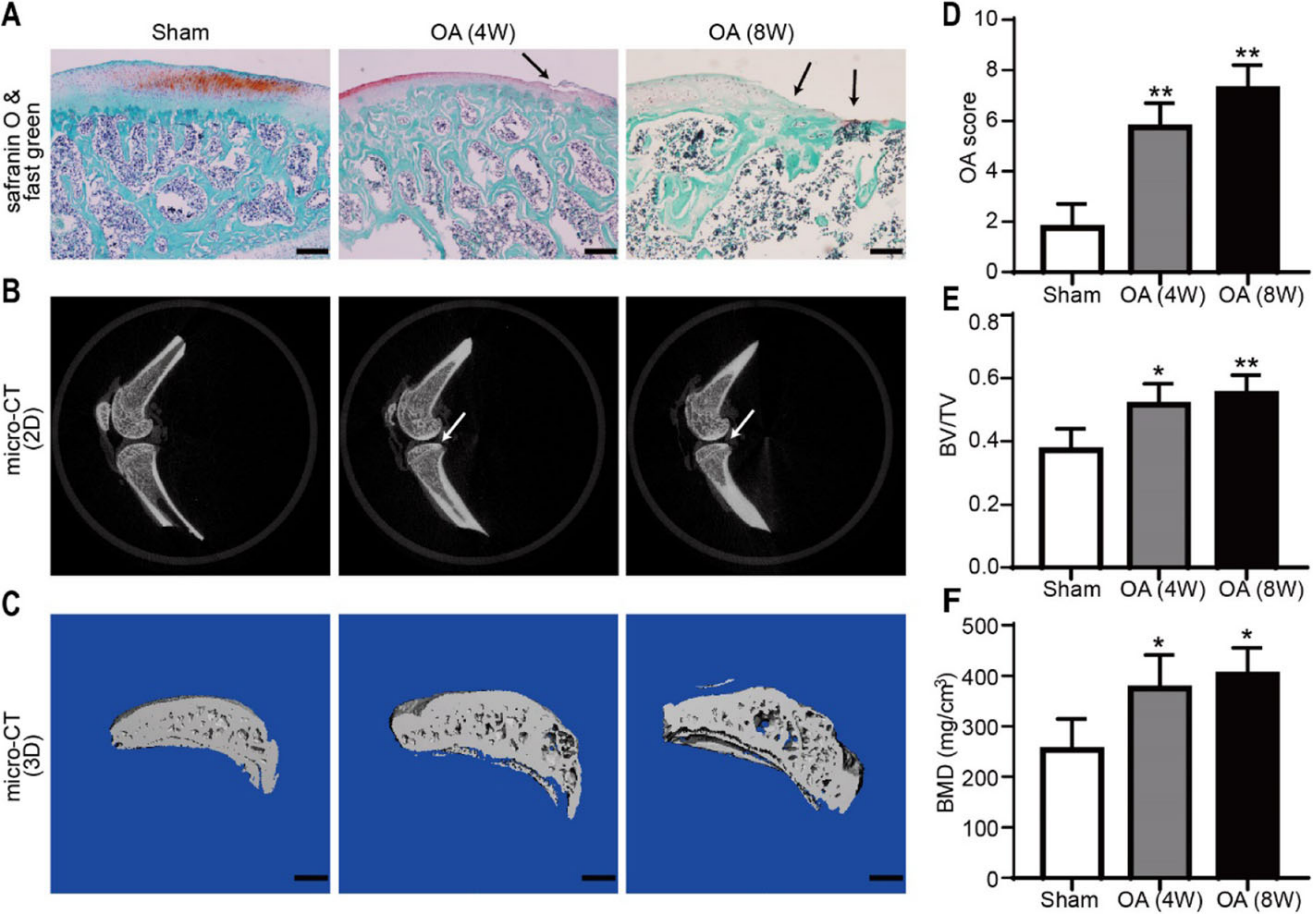
Histological and microstructural changes in the subchondral bone of the superior articular surface in OA model rats. Representative images from experiments are shown. **a** Safranin O/fast green-stained sections of the subchondral bone of the tibia. The damaged region of the articular surface is indicated by black arrows. Scale bar = 200 μm. **b** 2D imaging of the structure of the subchondral bone of the tibia. The damaged region of the articular surface is indicated by white arrows. **c** 3D imaging of the medial compartment of the subchondral bone of the tibia. Scale bar = 1.0 mm. **d** Quantitative results based on the OARSI scoring system. **e**, **f** Quantitative results of the structural parameters of the tibial subchondral bone, including BV/TV (trabecular BV per TV) and BMD (*n* = 8, *P < 0.05, **P < 0.01 vs sham)

Safranin O/fast green staining showed that the structure of the cartilage remained relatively intact in the sham-operated group, whereas the surface of the OA-group cartilage 4 and 8 weeks after operation was uneven with the disappearance of tide marks (Fig. 4a). The results based on the OARSI scoring system indicated that the cartilage destruction of the OA 4-week (5.75 ± 0.83, *n* = 4) and 8-week (7.25 ± 0.82, *n* = 4) groups was severe compared with that of the sham group (0.75 ± 0.43, *n* = 4; Fig. 4d). The 2D images and reconstructed 3D images obtained from the micro-CT were used to assess the microarchitecture of the rat subchondral bone in all groups and showed that the subchondral bone was hardened in the OA group compared with the sham group (Fig. 4b, c). The respective values were 0.51 ± 0.06 and 0.55 ± 0.05 vs 0.37 ± 0.06 for the BV/TV and 375.40 ± 57.95 mg/cm^3^ and 402.95 ± 46.08 mg/cm^3^ vs 253.45 ± 53.11 mg/cm^3^ for the BMD (*P* < 0.05, *n* = 3; Fig. 4e, f).

### Inflammation of the synovium in an OA animal model

**Fig. 5:**
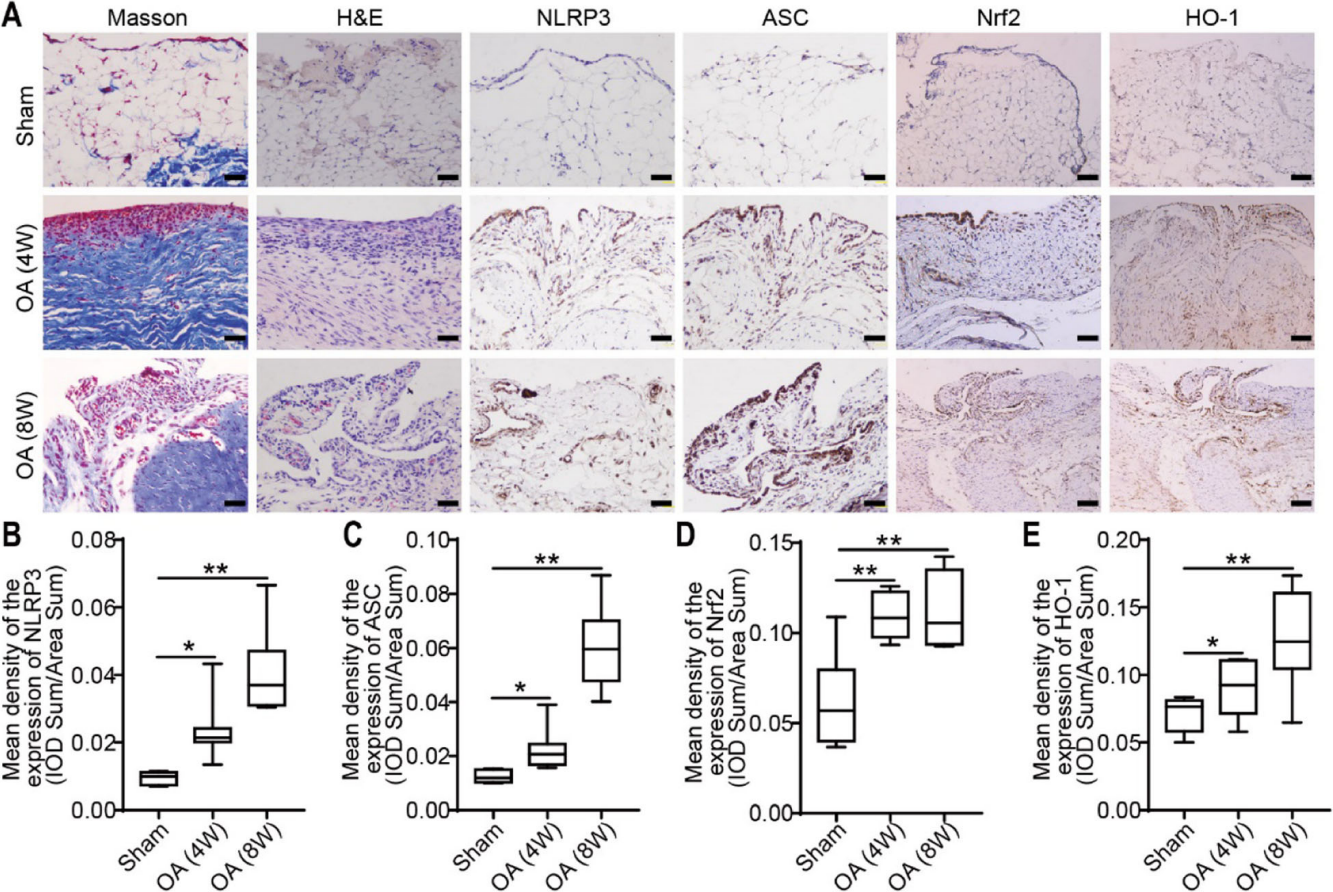
Histology and IHC staining of the synovium in OA model rats. **a** Histochemistry and IHC staining of the synovium from the sham, 4-week and 8-week OA groups. Representative images are shown above. Scale bar = 100 μm. **b** Relative expression level of NLRP3. **c** Relative expression level of ASC. **d** Relative expression level of Nrf2. **e** Relative expression level of HO-1 (*n* = 8, *P < 0.05, **P < 0.01 vs sham)

The synovia of the knee joints from the OA model rats were harvested for Masson and H&E staining 4 or 8 weeks after the operation. Compared with the sham group, the 4-week and 8-week OA groups displayed pathological changes in the synovium and cell infiltration. The lesions of the 8-week OA group were the most severe. IHC staining showed that the expression of NLRP3, ASC, Nrf2, and HO-1 in the synovial tissue of the OA group was significantly higher than that of the sham group. Nrf2, HO-1, NLRP3, and ASC expression in the synovium and the phenotype of the synovium were investigated in OA model rats by histochemistry. Colored staining, representing the expression of Nrf2, HO-1, NLRP3, and ASC, was observed in the synovium of OA rats (Fig. 5a). Compared with that in the sham group, the expression of NLRP3, ASC, Nrf2, and HO-1 in the 4-week OA group was increased (Fig. 5b-e). Notably, the expression of Nrf2 and HO-1 in the OA 8-week group was increased significantly compared with that in the sham group (Fig. 5d, e).

### Expression of ROS in SW982 cells treated with LPS

**Fig. 6:**
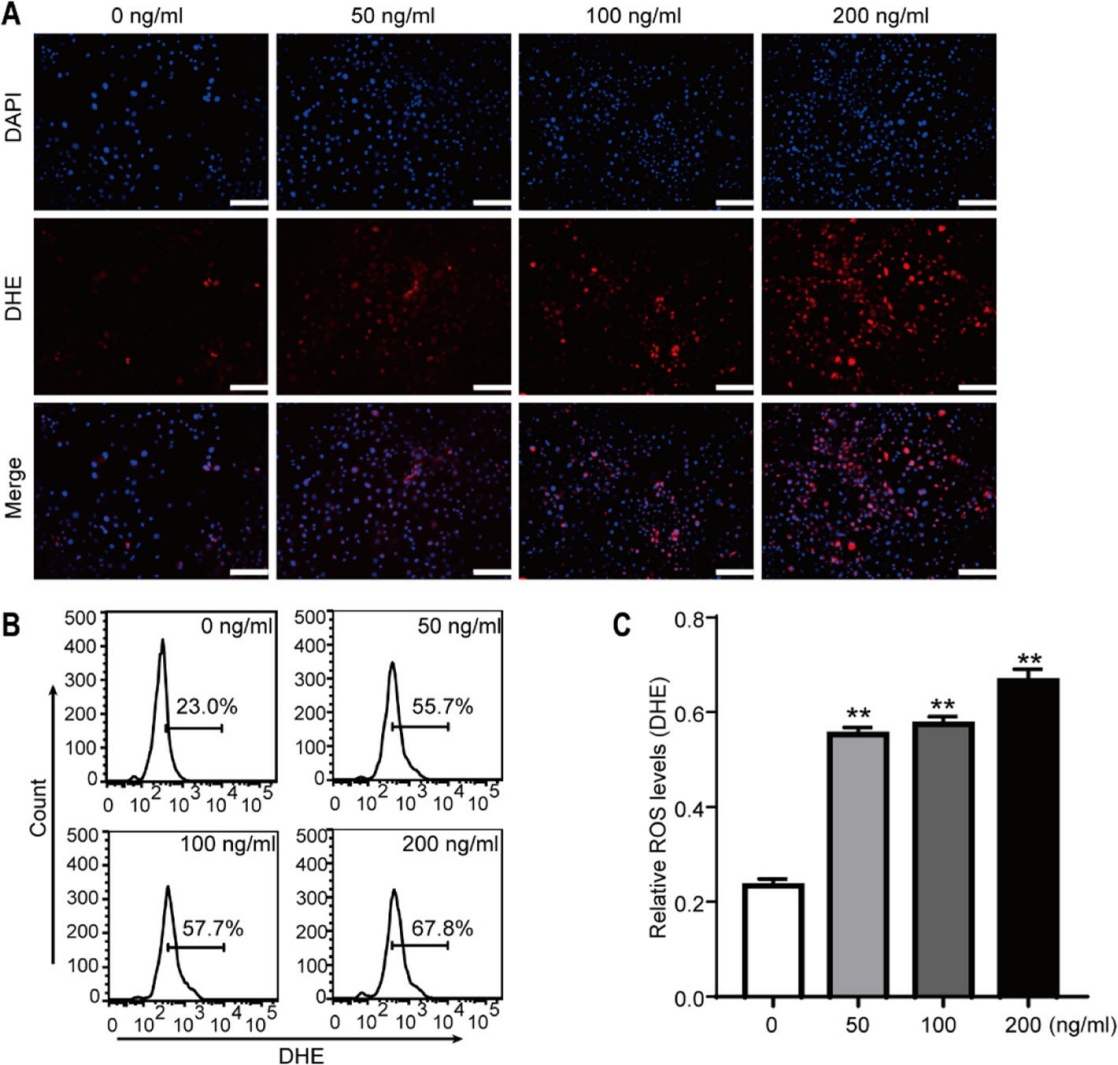
LPS treatment increases ROS generation in SW982 cells. **a** DHE staining (red fluorescence) was performed to evaluate the ROS levels. Representative images are shown from repeated experiments. Scale bar = 100 μm. **b** Flow cytometric analysis of the generation of ROS in cells treated with different concentrations of LPS for 24 h. **c** Relative levels of ROS (*n* = 3, **P < 0.01 vs CTL)

LPS treatment increased the inducible ROS production in SW982 cells. The results are shown in Fig. 6. ROS levels were assessed by DHE staining, and LPS treatment promoted the expression of ROS in a dose-dependent manner. Furthermore, a flow cytometric analysis was also performed for further confirmation of this finding.

### LPS-induced inflammation associated with ROS

**Fig. 7:**
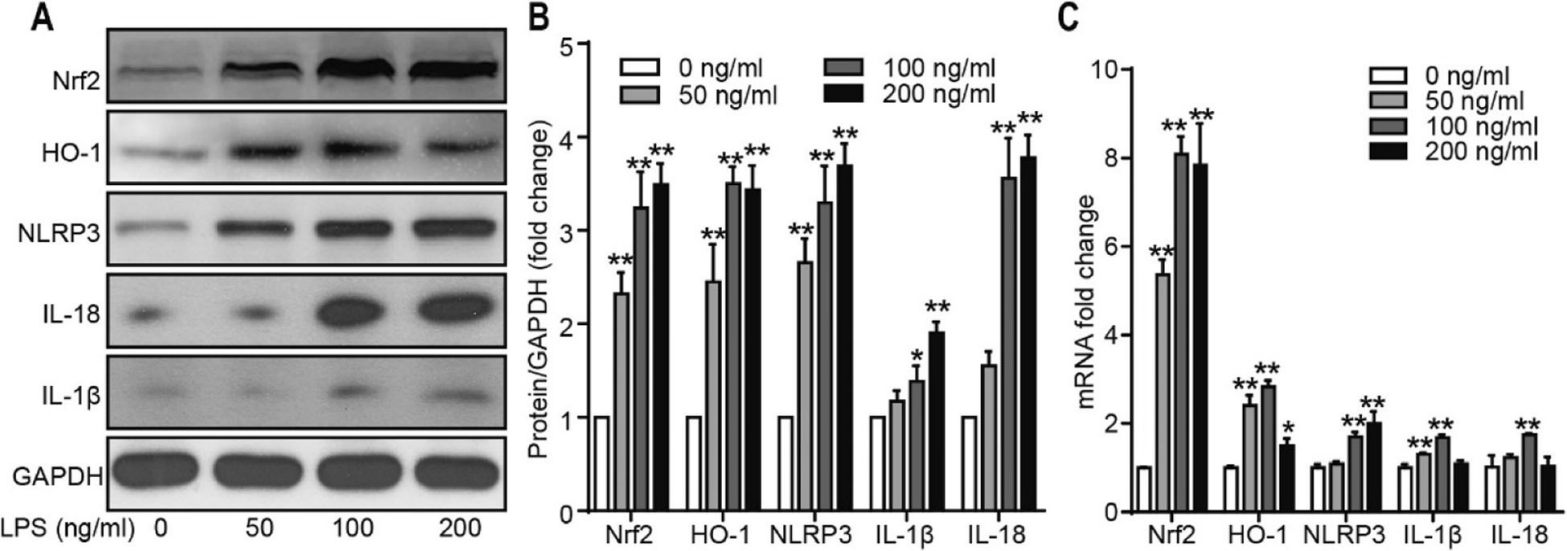
ROS-associated inflammation is induced in SW982 cells by treatment with LPS for 24 h. **a**, **b** Representative images of Western blots depicting the levels of NLRP3, Nrf2, IL-1β, and IL-18 in SW982 cells and the protein/GAPDH ratios as determined by the densitometric analysis of Western blots. **c** NLRP3, IL-1β, IL-18, Nrf2, and HO-1 mRNA expression examined using qRT-PCR (*n* = 3, *P < 0.05, **P < 0.01 vs CTL)

NLRP3 is a protein complex that is associated with ROS-induced inflammation. To understand the activation mechanism of the NLRP3 inflammasome, we studied the expression of related proteins and mRNAs in SW982 cells after stimulation with different concentrations of LPS. As shown in Fig. 7, the Western blot and qRT-PCR results indicated that the expression of Nrf2, HO-1, NLRP3, IL-1β, and IL-18 was upregulated by LPS treatment in a dose-dependent manner.

### Inflammation response to NLRP3 knockdown in SW982 cells

**Fig. 8:**
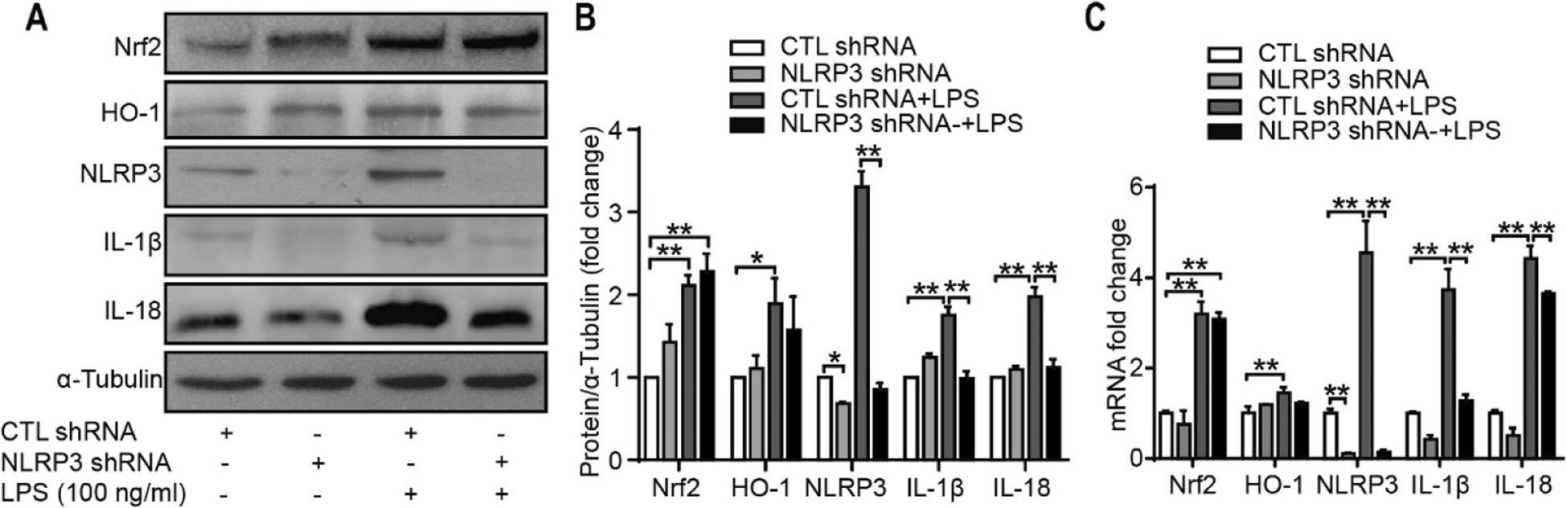
Expression of the proteins and related mRNAs involved in inflammation after treatment with LPS for 24 h. **a**, **b** Representative images of Western blots depicting the levels of NLRP3, Nrf2, IL-1β, and IL-18 in SW982 cells, and protein/α-tubulin ratios, as determined by the densitometric analysis of Western blots. **c** mRNA fold changes of Nrf2, HO-1, NLRP3, IL-1β, and IL-18, examined using qRT-PCR (*n* = 3, *P < 0.05, **P < 0.01 vs CTL shRNA)

To evaluate the roles of the NLRP3 inflammasome, cells were infected with lentiviral particles containing shRNA specific to NLRP3 and then stimulated with LPS for 24 h. The Western blot and qRT-PCR results showed that the expression of IL-1β and IL-18 was downregulated significantly. As shown in Fig. 8, NLRP3 shRNA significantly reduced the protein and mRNA levels of NLRP3, IL-1β, and IL-18. However, the protein and mRNA levels of Nrf2 and HO-1 did not change dramatically with or without LPS stimulation. These results suggest that the NLRP3 inflammasome is vital for LPS-induced IL-1β and IL-18 expression in SW982 cells. In addition, each component of the NLRP3 inflammasome is involved in IL-1β and IL-18 production.

### Inhibition of the Nrf2 pathway alleviates the inflammation induced by NLRP3 in response to LPS treatment

**Fig. 9:**
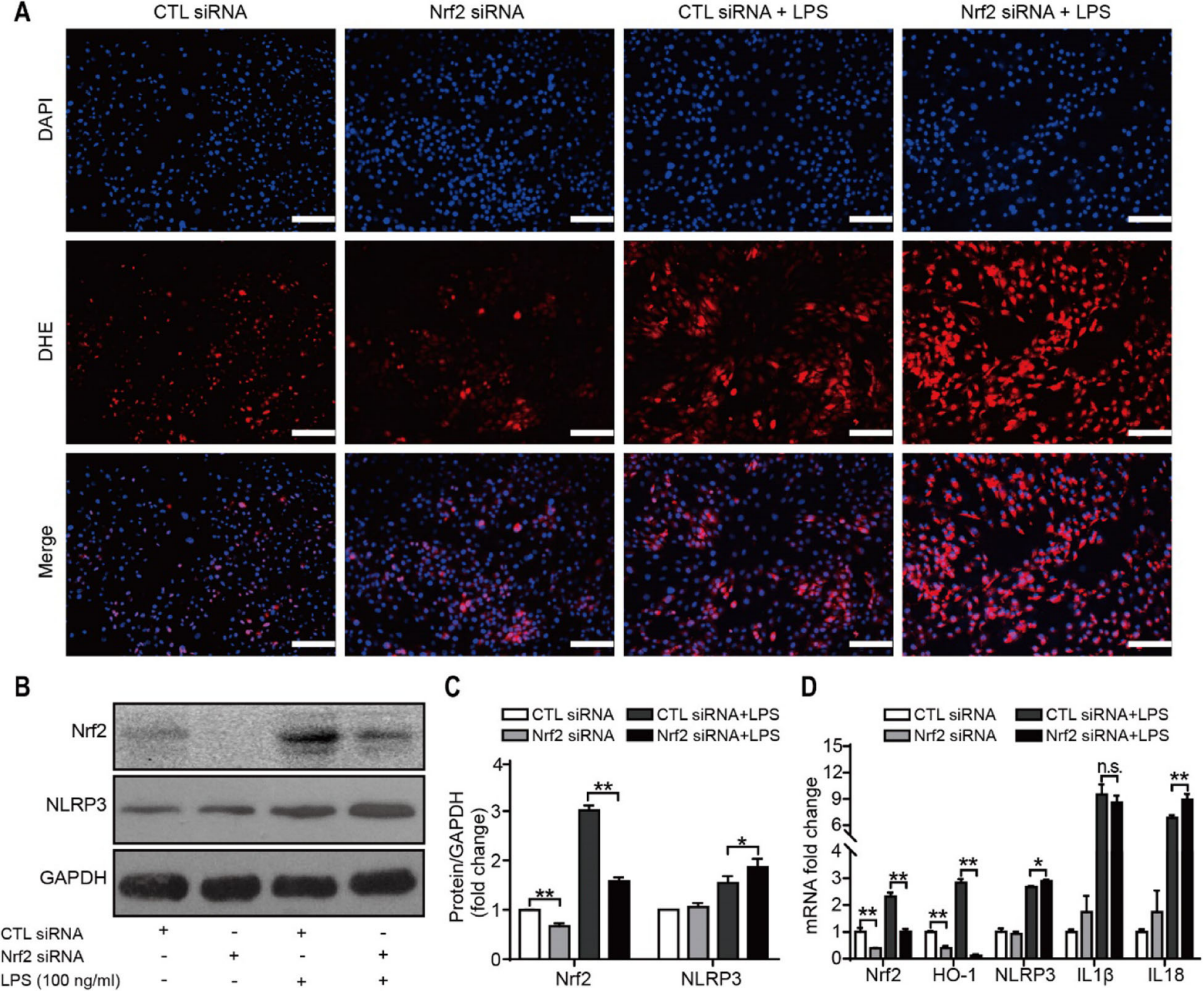
The expression of inflammation-associated proteins and mRNAs in Nrf2-knockdown SW982 cells treated with LPS for 24 h. **a** DHE staining (red fluorescence) was performed to evaluate the level of ROS. Representative images are shown from repeated experiments. Scale bar = 100 μm. **b**, **c** Representative images of Western blots depicting the expression levels of NLRP3 and Nrf2 in SW982 cells and the protein/GAPDH ratios as determined by the densitometric analysis of the Western blots. **d** Nrf2, HO-1, NLRP3, IL-1β, and IL-18 mRNA expression examined using qRT-PCR (*n* = 3, ^n.s.^*P* > 0.05, *P < 0.05, **P < 0.01)

To identify the relationship between the NLRP3 inflammasome and Nrf2, we transfected SW982 cells with siRNA specific for Nrf2 and stimulated them with LPS for 24 h. As shown in Fig. 9a, no significant difference was observed in the ROS level between cells treated with control siRNA and siNrf2, as assessed by DHE staining. However, the LPS-induced ROS production was enhanced after silencing of Nrf2. Furthermore, the downregulation of Nrf2 expression increased the expression of NLRP3 (Fig. 9b–d), which means that LPS-induced NLRP3 inflammasome activation may be enhanced after Nrf2 silencing.

## Discussion

Articular cartilage, the subchondral bone and the synovium are pivotal in maintaining the stability of the joint. Degeneration of the cartilage and subchondral bone plays a key role in the progression of OA. The synovium, which functions as a part of the lubrication, nutrition, and circulation system of the synovial fluid of the joint, plays an important role in maintaining the homeostasis of the joint. Furthermore, it contributes to the degeneration of the joint in OA by releasing inflammatory molecules [4, 18]. Evidence suggests that the NLRP3 inflammasome is involved in the development of OA, leading to the degradation of cartilage and synovial inflammation [8, 18]. In this study, we found that NLRP3 and ASC were highly expressed in the synovia of OA patients, as well as in the OA model rats, which is consistent with previous studies [19–21]. Moreover, several studies have confirmed that inflammasomes promote the expression and maturation of proinflammatory cytokines, including IL-1β and IL-18 [22, 23]. As well-documented inflammatory cytokines, IL-1β and IL-18 have been shown to be involved in the occurrence and development of various diseases, including OA [24, 25]. A previous study investigating the secretion of IL-1 β and IL-18 by knee joint articular cartilage and synovium explants from OA patients demonstrated that the release of those two cytokines from synovium was remarkably greater than that from articular cartilage [26], implying under pathological conditions, IL-1 β and IL-18 may be mainly from immune cells in synovium, rather than chondrocytes in cartilage. Meanwhile, results obtained from a study of hand osteoarthritis (HOA) showed NLRP3 protein expression in the peripheral blood mononuclear cells derived from nonerosive HOA patients was much higher compared to that from erosive HOA patients and healthy subjects [27]. Although the exact reason for this is still unknown, this finding is well in line with our current result showing enhanced NLRP3 expression in knee OA. It should be noted, however, that the role of NLRP3 in OA pathogenesis seems highly controversial. Indeed, while some studies suggested a contributing impact of NLRP3 on OA [28–30], others indicated that NLRP3 was not essential for cartilage degradation in OA [26, 31]. These conflicting results regarding NLRP3 may result from species difference in experimental OA models and variability in the method of OA induction.

ROS exist as both injurious and salutary species and play important roles in various physiological processes at low concentrations; however, the overproduction of ROS in cells leads to the onset and progression of OA [32]. It has been reported that ROS play an essential role in proinflammatory responses, affecting the activity of antioxidant enzymes and oxidative stress, leading to the activation of the NLRP3 inflammasome [28, 33, 34]. Accordingly, our study provides novel evidence suggesting that the expression level of Nrf2 and its downstream factor HO-1 is upregulated in the synovia of OA patients, which indicates that Nrf2, HO-1, and the NLRP3 inflammasome may play a crucial role in the progression of OA.

Nrf2 is an oxidative stress sensor and key transcription factor that protects cells from foreign substances and oxidative damage. Previous studies have shown that under physiological circumstances, Nrf2 is mainly present in the cytoplasm; however, when the level of ROS is elevated, the transcription of antioxidative stress proteins, including HO-1, is enhanced by Nrf2 [35, 36]. In addition, evidence suggests that the oxidative stress response transcription factor Nrf2, which is responsible for the induction of the transcription of major antioxidant enzymes, is required for the activation of cholesterol crystal-induced NLRP3 activation [37]. To better understand the relationship between NLRP3 and the Nrf2/HO-1 pathway, Nrf2-knockdown SW982 cells were treated with LPS. As shown in Fig. 9b, c, NLRP3 expression was significantly upregulated in the Nrf2-knockdown group compared with the siRNA control group. In general, Nrf2 activation is considered to have anti-inflammatory effects via the regulation of genes encoding proinflammatory cytokines that are involved in inflammation [38]. Our study results suggest that the activation of the NLRP3 inflammasome is induced by ROS after stimulation with LPS, and changes in ROS levels provide a direct interpretation of the anti-inflammatory activity of the Nrf2/HO-1 signaling pathway.

Additionally, the upregulation of HO-1 expression is important for cell protection under oxidative stress and inflammation conditions. A recent study reported that antioxidant proteins expression is increased after the activation of the Nrf2/HO-1 pathway, which in turn reduces inflammation and oxidative stress [39]. The heat shock protein family member HO-1 is an important anti-inflammatory, antioxidative, and cytoprotective enzyme that is regulated by the activation of the major transcription factor Nrf2 [40, 41]. Wu et al. [42] showed that Nrf2 activation and HO-1 induction exert anti-inflammatory effects and ROS inhibition. Consistent with this finding, as shown in Fig. 9a, there was no significant difference in the basal ROS levels between control siRNA- and siNrf2-treated cells, as assessed by using DHE staining; however, the LPS-induced level of ROS was enhanced in siNrf2-treated cells compared with control siRNA-treated cells.

Although we found that Nrf2, HO-1, NLRP3, and ASC were expressed in the knee synovia of patients with osteoarthritis, concrete evidence for the relationship between these genes and OA is still missing. In addition, due to the limitations of this study, the exact relationship between the synovium and the articular cartilage has not been clearly illustrated. Among inflammatory models, the ACLT+MMD rat model is of particular interest since it reproduces the main symptoms of OA at both the clinical and biological levels. However, this model does not completely simulate the entire process of OA in humans because it is a degenerative disease rather than a trauma-induced disease in most circumstances. Moreover, the cellular response to the inhibition of the Nrf2/HO-1 pathway is still unclear, even though the results of our study suggest that the NLRP3 inflammasome is activated by the ROS pathway in vivo.

## Conclusions

**Fig. 10:**
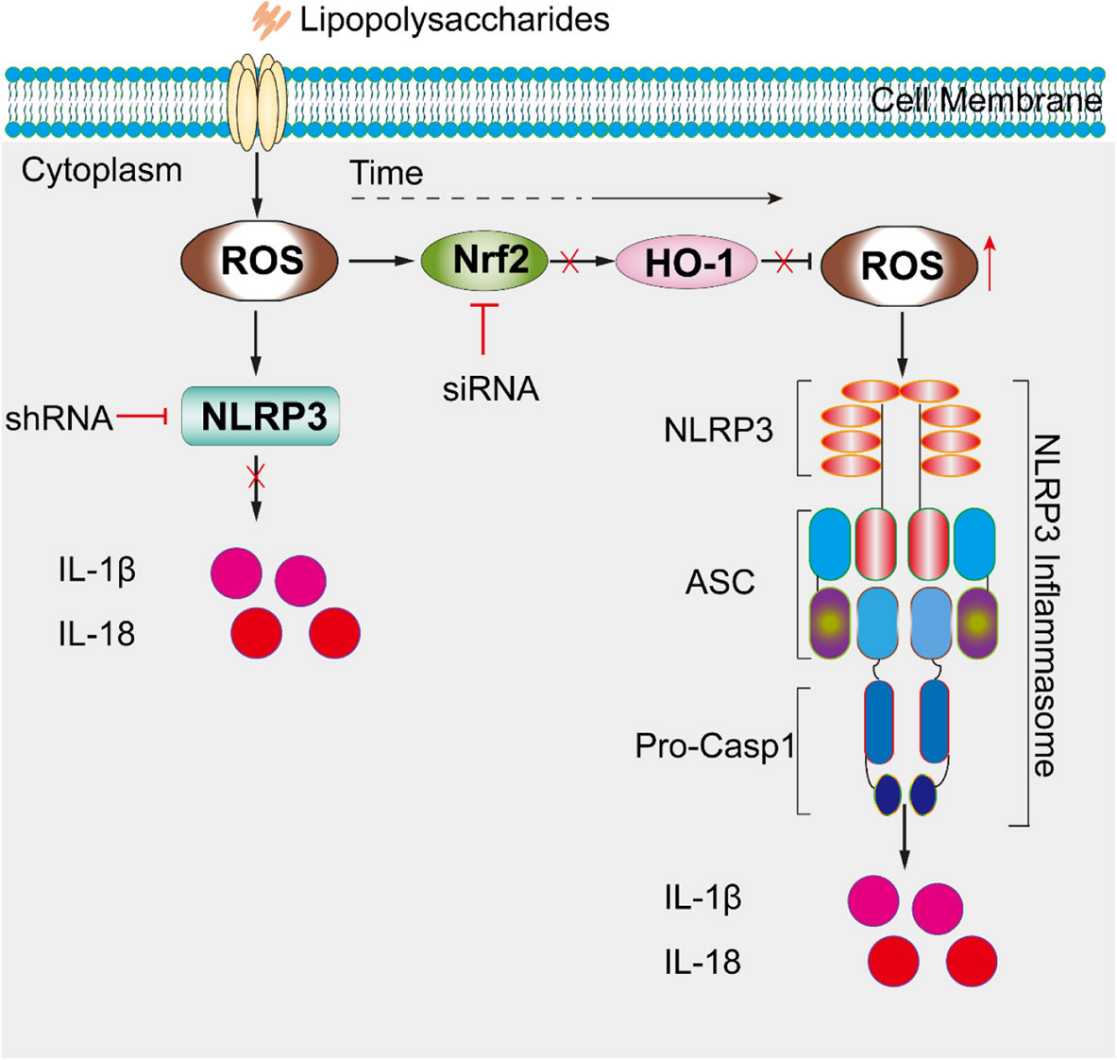
The proposed pathway of NLRP3 inflammasome activation in SW982 cells stimulated with LPS

Taken together, the findings of our study demonstrate that oxidative stress leads to the activation of the NLRP3 inflammasome. Nrf2, HO-1, NLRP3, and ASC are highly expressed in the synovia of OA patients and OA model rats. Furthermore, we found reducing Nrf2 expression leads to the upregulation of NLRP3 expression in vitro, as illustrated in Fig. 10, which may be a pivotal process in the development of OA. Therefore, it is reasonable to speculate that targeting Nrf2 signaling might be a promising and attractive therapeutic strategy to prevent OA.

## Data Availability

The datasets analyzed during the current study are available from the corresponding author on reasonable request.

## Abbreviations

ASC: Apoptosis-associated speck-like protein containing a CARD
ATP: Adenosine triphosphate
BMD: Bone mineral density
BV: Bone volume
CT: Computed tomography
DHE: Dihydroethidium
DMEM: Dulbecco’s modified Eagle’s medium
EDTA: Ethylenediaminetetraacetic acid
ELISA: Enzyme-linked immunosorbent assay
FBS: Fetal bovine serum
GAPDH: Glyceraldehyde 3-phosphate dehydrogenase
H&E: Hematoxylin and eosin
HBSS: Hank’s balanced salt solution
HO-1: Heme oxygenase 1
IHC: Immunohistochemistry
IL: Interleukin
LPS: Lipopolysaccharide
NADPH: Nicotinamide adenine dinucleotide phosphate
NLRP3: Nucleotide-binding and oligomerization domain-like receptor containing protein 3
Nrf2: Nuclear factor-erythrocyte 2 related factor 2
OA: Osteoarthritis
OARSI: Osteoarthritis Research Society International
PVDF: Polyvinylidene fluoride
ROS: Reactive oxygen species
SD: Sprague-Dawley
SDS-PAGE: SDS-polyacrylamide gel electrophoresis
TBS-T: Tris-buffered saline-Tween 20
TV: Tissue volume
